# Detection of *invA* virulence gene of multidrug-resistant *Salmonella* species isolated from the cloacal swab of broiler chickens in Blitar district, East Java, Indonesia

**DOI:** 10.14202/vetworld.2021.3126-3131

**Published:** 2021-12-19

**Authors:** Freshindy Marissa Wibisono, Hayyun Durrotul Faridah, Freshinta Jellia Wibisono, Wiwiek Tyasningsih, Mustofa Helmi Effendi, Adiana Mutamsari Witaningrum, Emmanuel Nnabuike Ugbo

**Affiliations:** 1 Department of Veterinary Public Health, Faculty of Veterinary Medicine, Universitas Airlangga, Surabaya, Indonesia; 2 Department of Biology, Faculty of Science and Technology, Universitas Airlangga, Surabaya, Indonesia; 3 Department of Veterinary Public Health, Faculty of Veterinary Medicine, Wijaya Kusuma University Surabaya, Surabaya, Indonesia; 4 Department of Veterinary Microbiology, Faculty of Veterinary Medicine, Universitas Airlangga, Surabaya, Indonesia; 5 Department of Veterinary Public Health, Faculty of Veterinary Medicine, Universitas Airlangga, Surabaya, Indonesia; 6 Department of Applied Microbiology, Ebonyi State University, Abakaliki, Nigeria

**Keywords:** broiler chicken, *inv*A gene, multidrug-resistant, public health, *Salmonella*

## Abstract

**Background and Aim:**

The increasing number of multidrug-resistant (MDR) *Salmonella* species on poultry farms in Indonesia has caused concern regarding human health. This study was conducted to determine the presence of the virulence gene *inv*A in MDR *Salmonella* species isolated from the cloacal swab of broiler chickens in Blitar district, East Java Province, Indonesia.

**Materials and Methods:**

Cloacal swab samples were collected by purposive sampling from 15 farms in four districts. Isolation and identification of bacteria were performed using standard microbiological techniques. Confirmation of MDR isolates was done using five different classes of antibiotics, including the beta-lactam, aminoglycoside, fluoroquinolone, phenicol, and monobactam groups. An antibiotic susceptibility test was conducted using the Kirby–Bauer disk diffusion method, and a polymerase chain reaction method was used to screen for the presence of *inv*A.

**Results:**

It was observed that 32.26% (50/155) of the samples were positive for *Salmonella* species. Of these 50 *Salmonella* isolates, 7 (14%) were identified as MDR strains. An important finding was the detection of *inv*A in all the seven MDR *Salmonella* strains (100%) isolated from the cloacal swab of broiler chickens in Blitar district, East Java Province.

**Conclusion:**

Veterinarians have an extremely important role in monitoring the use of antibiotics in farm animals to mitigate the rapid spread of MDR organisms in our environment, which can otherwise cause serious economic losses and also public health issues.

## Introduction

*Salmonella* species have been the major cause of foodborne diseases in several countries in the past 100 years [1-3]. It has been reported worldwide that there are 1.6 million cases of typhoid fever, 1.3 billion cases of gastroenteritis, and 3 million deaths due to *Salmonella* species [4]. Salmonellosis is a disease caused by the pathogenic agent *Salmonella* spp. Salmonellosis has been categorized as an important public health zoonosis [5-7] with a high morbidity rate and is difficult to prevent [8,9]. *Salmonella* species have several virulence factors that are important in the process of infection in the host and spread of disease. Most virulence factors are located in chromosomal genes known as *Salmonella* pathogenicity islands (SPIs). SPIs are essential for invasion and proliferation in host cells [10,11].

Chromosomal virulence genes comprise *inv*A, *inv*E, *him*A, and *pho*P, which are included among the target genes for polymerase chain reaction (PCR) amplification in *Salmonella* spp. [12,13]. *Salmonella* spp. harbor *inv*A that plays a role in causing illness. More than 50% of these serotypes are *Salmonella enterica*, which accounts for the majority of human *Salmonella* infections [3]. This gene is located in the area of SPI, which has an operon that functions as a repository for genetic information [14]. *inv*A from *Salmonella* spp. contains a unique DNA sequence and has been confirmed to be a suitable PCR target gene for the detection of *Salmonella* spp. [15]. This gene encodes proteins in bacterial cell membranes that are required for invasion into host epithelial cells [16]. *In vitro* amplification of DNA by the PCR method is an accurate tool for microbiological diagnosis [17].

Blitar has the largest chicken farms in East Java province, which consists of layer and broiler farms [18]. This high population enables the development of several diseases. The incidence of infection with *Salmonella* species is caused by contact with animals [9,19]. Infection can be acquired by both direct and indirect contact with animals [20]. Due to the increasing incidence of infectious diseases, antibiotic use has become the most predominant strategy in health services [21,22]. Such high use of antibiotics has resulted in an increase in antibiotic resistance [23].

This study was conducted to determine the presence of the virulence gene *in*vA in multidrug-resistant (MDR) *Salmonell*a species isolated from the cloacal swab of broiler chickens in Blitar district, East Java Province, Indonesia.

## Materials and Methods

### Ethical approval

Ethical approval was not necessary. However, cloacal swab samples were collected as per the standard collections method without any harm to the broiler.

### Study period and location

This research was conducted from October 2020 to February 2021. Samples were collected from 15 broiler farms in Blitar district. The farms are located in four sub-districts, namely Ponggok, Garum, Selopuro, and Selorejo sub-districts. Samples were processed at Laboratory of Department of Veterinary Public Health, Faculty of Veterinary Medicine, Universitas Airlangga.

### Sample collection

**Figure-1:**
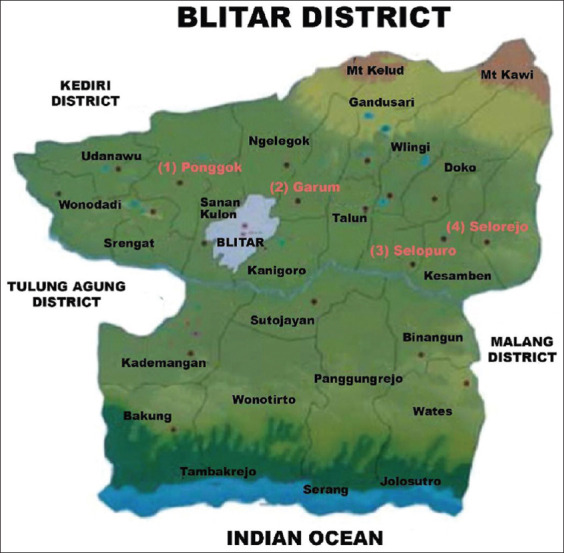
Map of the distribution of sampling in Blitar district. (1) Ponggok subdistrict; (2) Garum subdistrict; (3) Selopuro subdistrict; and (4) Selorejo subdistrict [25].

The sampling technique was carried out by purposive sampling from broiler farms that had been determined by the researchers with criteria, namely, information from the relevant agencies, and broiler chickens that had clinical digestive symptoms such as diarrhea, abnormal stool color, weakness, and dirty parts around the cloaca. Samples were taken from the cloacal swab of broiler chickens in Blitar district [24]. The sample size used was 155 cloacal swab samples from 15 broiler farms on four subdistricts (Figure-1) [25].

### Bacterial isolates

**Figure-2:**
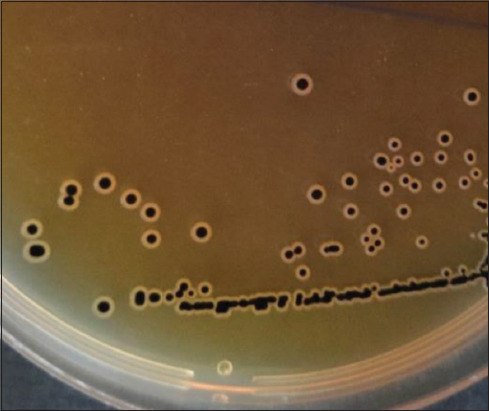
Pure colonies of *Salmonella* spp. on SSA media.

Bacterial testing included isolation and identification of *Salmonella* isolates. Bacterial isolation was conducted by collecting a suspension using an inoculation loop sterilized under a Bunsen burner fire. The suspension was implanted in Salmonella–Shigella agar (Figure-2). *Salmonella* growth produces a transparent or colorless colony with a black center due to the formation of H_2_S gas. Presumptive *Salmonella* was placed on bismuth sulfite agar media. Bismuth sulfite is a selective medium for the isolation of *Salmonella* in the laboratory and is generally used for the detection of *Salmonella* species.

### Antibiotic sensitivity test

All *Salmonella* spp. isolates were subjected to the antibiotic sensitivity test, and confirmation of MDR isolates was done using antibiotic sensitivity tests to five different classes of antibiotics. The following antibiotic disks (Oxoid, England) were used: Beta-lactam (amoxicillin 10 g), aminoglycoside (gentamicin 10 g), quinolone (ciprofloxacin 5 g), phenicol (chloramphenicol 30 g), and monobactam (aztreonam 30 g) groups.

### PCR amplification of *invA*

All the MDR *Salmonella* spp. were tested for the presence of the virulence gene *inv*A [26,27]. This gene is responsible for the virulence factor of *Salmonella* spp. The specific primers used to detect *inv*A in *Salmonella* isolates have been previously published [16], which included the forward primer 5́ GTG AAA TTA TCG CCA CGT TCG GGC AA 3́ and the reverse primer 5́ TCA TCG CAC CGT CAA AGG AAC C 3́ (284 bp). This study was conducted at the Veterinary Public Health Laboratory, Faculty of Veterinary Medicine, Universitas Airlangga, and the Microbiology Laboratory of the Central Health Laboratory, Surabaya. In the PCR analysis, *Escherichia coli* isolate ATCC 25922 was used as a negative control, and *Salmonella* Paratyphi A isolate ATCC 9150 was used as a positive control, which had been previously tested for the presence of *inv*A.

## Results and Discussion

*Salmonella* species remains one of the major causes of foodborne diseases such as salmonellosis and other related diarrhea in several countries in the world [3]. The findings of this study indicated that 32.26% (50/155) of the samples were positive for *Salmonella* isolates (Table-1). The highest incidence occurred in Garum subdistrict (60.00%), followed by Ponggok (28.30%) and Selopuro (27.45%) subdistricts, and the lowest incidence occurred in Selorejo subdistrict (23.08%). These results indicated the presence of high infection with *Salmonella* spp. in Blitar district. The overall incidence is much higher than that of the previous studies, which reported an incidence of 3.2% in layer chickens and 12.4% in broiler chickens from five provinces in Java [28]; however, the incidence of salmonellosis in layer chicken farms in Sidrap district was 76.39% [29]. Our study findings indicate the need for increased attention toward an immediate follow-up by relevant policymakers as an effort to prevent the spread of zoonotic diseases [30]. Salmonellosis is a zoonotic disease caused by *Salmonella* spp., which can affect humans and animals. Indirect transmission can occur through contact with the environment around animals or with contaminated objects around poultry farms [6,31].

**Table 1:** Isolation and identification of *Salmonella* species on Blitar district.

Subdistrict	Samples	*Salmonella* spp.	Percentage
Ponggok	53	15	28.30
Garum	25	15	60.00
Selopuro	51	14	27.45
Selorejo	26	6	23.08
Total	155	50	32.26

The high incidence of salmonellosis in Garum subdistrict compared to that in the other three subdistricts is due to the relatively low sanitation in poultry farms. In general, the farm is located behind the farmer’s house. Field conditions showed the sewerage from the farmer’s house is adjacent to the cage. Contamination and infection with *Salmonella* spp. on a farm with poor sanitation can occur due to the spread of *Salmonella* spp. through contaminated feces, thereby contaminating feed, drinking water, and hatching eggshells [28].

Salmonellosis can be treated using antibiotics. The emergence of MDR *Salmonella* species has now received the attention of various researchers. MDR indicates the resistance of bacteria to three or more classes of antibiotics [32]. Such resistance to some of these antibiotics can occur due to the pattern of continuous use of antibiotics in the livestock industry as both treatment and feed additives and growth promoters. The continuous use of antibiotics is a triggering factor for high levels of antibiotic resistance [33,34].

In this study, quinolone and beta-lactam exhibited the highest yields of 46% and 44%, respectively (Table-2). This is because these classes of antibiotics are often used in broiler farms in Blitar district. Fluoroquinolones have a very narrow safety range and are safe in low or high doses but for a short time. Continuous use for prolonged periods can cause side effects [35]. Beta-lactams are the most widely used class of antibiotics in both human clinical practice and veterinary medicine because they are broad-spectrum antibiotics and exhibit a very good level of safety [34]. Routine administration of broad-spectrum antibiotics is one of the supporting factors for the changing patterns of resistance to various antibiotics [36]. Cases of antibiotic resistance in bacteria can be caused by a lack of supervision on the use of antibiotics in farms, as 72.3% of farmers use antibiotics without veterinary supervision [37]. Risk factors such as the broiler breed, breeder education, type of partnership business, type of factory feed, veterinarian support in livestock rearing management, cage hygiene sanitation, chlorine treatment of drinking water, the existence of an antibiotic program, and reference to the use of antibiotics are positively associated with the incidence of MDR in chicken farms [38,39].

**Table 2:** Multidrug resistance of *Salmonella* spp. on broiler chicken.

Location	*Salmonella* spp.	Beta-lactam		Aminoglycoside		Quinolone		Phenicol		Monobactam		Multidrug resistant	
					
		R	%	R	%	R	%	R	%	R	%	Total	%
Ponggok	15	5	33.3	4	26.7	3	20	2	13.3	0	0	0	0.00
Garum	15	5	33.3	2	13.3	6	40	2	13.3	0	0	3	20.00
Selopuro	14	8	57.1	4	28.6	11	78.6	0	0	0	0	3	21.43
Selorejo	6	4	66.7	2	33.3	3	50	2	33.3	0	0	1	16.67
Total	50	22	44	12	24	23	46	6	12	0	0	7	14

The findings of this study showed that seven Salmonella isolates were MDR) (Table-2), as they were resistant to three different classes of antibiotics. MDR is caused by the continuous use of one type of antibiotic by the farmers. However, no single isolate was collectively resistant to the same antibiotic class. The existence of a rotation program in the administration of antibiotics can prevent the incidence of antibiotic resistance. Hence, it is necessary to educate farmers on the use of different antibiotics or the rotation of antibiotics during disease treatment. The role of veterinarians is also extremely important in monitoring the use of antibiotics on farms. Antibiotics must be used wisely and in the correct dose, and not as a disease prevention strategy [38,40,41].

**Figure-3:**
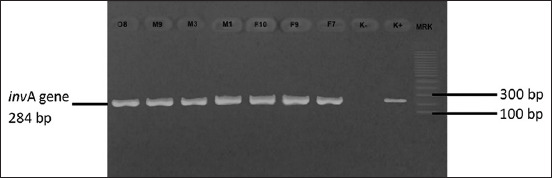
Gene *invA* of *Salmonella* spp. multidrug-resistant isolates. K+=*Salmonella* Paratyphi A ATCC 9150 as positive control; K–=*E. coli* ATCC 25922 as negative control; F7, F9, F10, M1, M3, M9, and O8=Multidrug-resistant isolates.

Our results also confirmed the presence of the virulence gene *inv*A in all the seven (100%) MDR *Salmonella* isolates using *inv*A-specific primers (Figure-3). In all these isolates, *inv*A was successfully amplified at 284 bp. This finding indicated that it is difficult to treat salmonellosis caused by MDR isolates because they have the ability to be pathogenic as evidenced by the presence of *inv*A. This gene encodes membrane proteins in bacteria that are responsible for invading host intestinal cells [42,43]. In *Salmonella* isolates, *inv*A was located in SPI-1, which plays an extremely important role in the invasion of host epithelial cells. This gene is highly specific for most *Salmonella* species [44,45]. *Salmonella* spp. harbors numerous virulence factors, which is one of the causes for the high incidence of salmonellosis in humans and animals. Intensive use of antibiotics for salmonellosis treatment leads to the emergence of resistant bacteria. One of the best assays for identifying virulence genes is PCR [46,47].

## Conclusion

In total, 32.26% (50/155) of the samples were positive for *Salmonella* species. Of the 50 isolates, 7 (14%) were found to be MDR. The virulence gene *inv*A was identified in all the seven MDR *Salmonella* strains (100%) isolated from the cloacal swab of broiler chickens in Blitar district, East Java Province. Veterinarians have an extremely important role in monitoring the use of antibiotics in farm animals to mitigate the rapid spread of MDR organisms in our environment, which can otherwise cause serious economic losses and also public health issues.

## Authors’ Contributions

MHE, FMW, and FJW: Conceptualization. MHE, FMW, and HDF: Data curation. WT and AMW: Formal analysis. MHE and WT: Funding acquisition. FMW, HDF, and FJW: Investigation. MHE and AMW: Methodology. MHE and AMW: Project administration. MHE, FMW, and FJW: Resources. MHE and WT: Supervision. MHE, WT, and ENU: Validation. FMW and AMW: Visualization. MHE, FMW, and FJW: Writing original draft. MHE and ENU: Review and editing. All authors read and approved the final manuscript.
